# A T2-weighted MRI grading system for preoperative prediction of visceral pleural invasion in small non-small cell lung cancers

**DOI:** 10.3389/fonc.2025.1682929

**Published:** 2025-11-27

**Authors:** Ning Zhang, Bei Liu, Hui Feng, Yang Li, Xiaolong Gu, Qian Xu, Li Yang, Xinran Wang, Yingmin Zhai, Gaofeng Shi

**Affiliations:** Department of Radiology, The Fourth Hospital of Hebei Medical University, Shijiazhuang, China

**Keywords:** non-small cell lung cancer, visceral pleural invasion, T2-weighted magnetic resonance imaging, tumor staging, preoperative prediction, diagnostic accuracy

## Abstract

**Purpose:**

To evaluate the supplemental diagnostic value of T2WI for assessing VPI in NSCLC ≤3 cm with indeterminate CT features (pleural contact/tags).

**Materials and methods:**

This prospective single-center study enrolled 138 participants with NSCLC ≤3 cm and CT-suspected pleural involvement, of whom 98 underwent surgical resection and histopathologic VPI confirmation from January 2021 to March 2023. All participants underwent thoracic MRI (3.0T), including T2WI sequences. Two radiologists independently graded tumor-pleural interface signals (Grades 0-3: absent to wedge-shaped hyperintensity). Pathologic VPI served as the reference standard. Logistic regression and ROC analysis were performed to develop a predictive model integrating tumor size and T2WI grades.

**Results:**

VPI-positive lesions (35/98, 35.7%) demonstrated larger mean tumor size (22.32 ± 5.58 mm vs. 16.17 ± 4.22 mm; *P* = 0.024) and higher T2WI hyperintensity frequencies (85.71% vs. 52.38%; *P* = 0.001). Grade 3 T2WI signals (wedge-shaped) achieved 93.65% specificity and 76.47% PPV for VPI, with a positive likelihood ratio of 5.85. Multivariate analysis identified tumor size (OR = 1.126/mm, *P* = 0.024), Grade 2 (OR = 8.826, *P* = 0.003), and Grade 3 signals (OR = 29.890, *P* < 0.001) as independent VPI predictors. The combined model achieved an AUC of 0.837 (95% CI: 0.758–0.916), demonstrating superior diagnostic performance versus CT-based criteria.

**Conclusion:**

T2WI-based grading of tumor-pleural interface hyperintensity serves as a radiation-free method for preoperative VPI prediction in small NSCLC, offering potential as a complementary tool to CT diagnosis. While promising, these findings require multicenter validation due to the single-center design.

## Introduction

Non-small cell lung cancer (NSCLC) remains a leading cause of cancer-related mortality worldwide (1–3). Among the prognostic indicators of NSCLC, the anatomic extent of the disease—which is based on the TNM staging system—is the most important prognostic factor. The TNM stage affects diagnosis, treatment planning, and prognosis for this malignancy. Visceral pleural invasion (VPI) constitutes a critical prognostic determinant in NSCLC and a crucial predictor of postoperative recurrence and lymph node metastasis (4–7).The 8th edition of TNM staging standard for NSCLC suggests that cT1 lung cancer with VPI should be upgraded to cT2, and IA to IB (8). Patients who had tumors with VPI had worse disease-free and recurrence-free survival and a higher rate of local and distant disease recurrence (9–11). Accurately identifying VPI preoperatively is critical to guide surgical strategy (12), determine adjuvant treatment needs (13), and inform survival expectations (5).

Preoperative assessment of the presence or absence of VPI plays a significant role in formulating surgical plans and selecting postoperative adjuvant therapy (6, 12–14). A few studies have demonstrated promising results indicating that VPI can be predicted by using the following preoperative CT findings: (a) direct pleural contact of the tumor (15), (b) pleural retraction (16), and (c) pleural tags (17). However, the accuracy of CT features for pathological VPI prediction ranged from 62.7% (432 of 689 patients) to 72.3% (498 of 689 patients) the positive predictive values ranged from 44.1% (173 of 392 patients) to 56.4% (88 of 156 patients), suggesting that approximately half of the CT-based predictions were false-positive (18).

Magnetic resonance imaging (MRI) is potentially advantageous for VPI detection because of its superior soft tissue contrast and tissue characterization properties, without ionizing radiation exposure. Conventional MRI can yield high-quality images of the thorax, particularly with respiratory-gated techniques. Among thoracic MRI sequences, compared with other sequences (such as UTE), T2WI can provide great contrast and details for lung lesions. While MRI’s superior soft tissue contrast theoretically aids VPI detection, no study has systematically evaluated a qualitative T2WI signal grading system specifically for preoperative VPI prediction in NSCLC ≤3 cm with indeterminate CT features. This gap motivated our development of a four-tier grading system (Grades 0-3) targeting this clinical dilemma. Therefore, the purpose of this study was to evaluate whether T2WI could provide supplemental diagnostic value for VPI assessment in NSCLC ≤3 cm with indeterminate CT features (pleural contact/tags).

## Materials and methods

This prospective, single-center study was conducted at the Forth Hospital of Hebei Medical University. The study was approved by the local medical ethics committee (Approval No. 2023KS186), with written informed consent obtained from all participants. All procedures involving human subjects were in accordance with the 1964 Helsinki declaration and its later amendments. The manuscript was prepared and revised according to the STARD checklist of items (**Supplementary Table S1**).

### Study participants

This study specifically targeted the clinical scenario where CT findings of pleural contact/tags create diagnostic uncertainty - representing precisely those cases where supplemental MRI could meaningfully influence management decisions.

The study initially enrolled 138 patients with suspected NSCLC presenting pulmonary lesions ≤3 cm on CT imaging randomly, meeting ≥1 of the following criteria indicating potential pleural involvement from January 2021 to March 2023: (1) Direct pleural contact: Tumor-pleura interface length ≥3 mm on axial images. (2) Pleural tags: Linear soft-tissue strands extending from nodule to pleura. All patients underwent thoracic MRI within 1 week after CT scan. Exclusion criteria comprised: (1) cases with suboptimal MRI quality, (2) patients not undergoing surgery within 2 weeks after MRI scan, (3) non-NSCLC pathologies, (4) undocumented VPI status.

### CT scanning

All CT examinations were conducted using either of two CT scanners: a Somatom Definition Flash (Siemens Healthineers, Erlangen, Germany) or a uCT 780 (United Imaging Healthcare, Shanghai, China), operated by certified radiologic technologists. Standard thoracic CT protocols included the following technical parameters: 120 kVp tube voltage with automated tube current modulation (reference 80–120 mAs), 1.0 mm reconstructed slice thickness (1.0 mm interval reconstruction using medium-smooth kernel B70f), 340 mm field-of-view adjusted to individual patient anatomy, and 0.5 s/rotation gantry speed. All scans were performed in the supine position during end-inspiratory breath-hold using a tri-phasic injection protocol with iodinated contrast media (350 mgI/mL). The standardized scan coverage spanned from the supraclavicular fossa to the inferior diaphragmatic surface, ensuring inclusion of the entire thoracic cavity and upper abdominal compartments for comprehensive staging assessment.

### MRI scanning

All imaging examinations were conducted on a 3.0 Tesla MRI system (MAGNETOM Skyra, Siemens Healthineers, Erlangen, Germany) equipped with a 60-channel phased-array torso coil. Study participants were positioned supine with arms elevated above the head to minimize respiratory motion artifacts. Prior to scanning, each patient underwent standardized breathing instruction for approximately 5 minutes, performed by an experienced MRI technologist. The standardized imaging protocol comprised four essential sequences: (1) Axial T1-weighted turbo spin-echo (TSE) [TR/TE = 650/9.8 ms, slice thickness = 3 mm, gap = 0.6 mm, matrix = 320×256, FOV = 380×380 mm²]; (2) Axial T2-weighted TSE [TR/TE = 2500/79 ms, flip angle = 240°, slice configuration as above]; (3) Axial short tau inversion recovery (STIR) sequence[TR/TE/TI = 2500/79/240 ms, slice thickness = 3 mm, matrix = 320×320, identical FOV]; and (4) Diffusion-weighted imaging (DWI) employing multiple b-values (0, 800, 1500 s/mm²) using single-shot echo-planar readout [TR/TE = 3500/68 ms, parallel imaging factor = 2]. All acquisitions utilized respiratory triggering and were obtained in axial plane with identical geometric parameters unless otherwise specified.

### Pathological analysis

Pathological examinations were performed using elastic fiber staining to evaluate the degree of pleural invasion. All specimens were assessed by a board-certified pathologist with 10 years of experience in pulmonary histopathology, who categorized pleural invasion according to established criteria: PL0 (no pleural invasion); PL1 (tumor extension through the elastic layer of visceral pleura without reaching the pleural surface); PL2 (tumor involvement of the visceral pleural surface); and PL3 (tumor penetration into the parietal pleura). PL1–3 were identified as VPI (6).

### Imaging analysis

**Figure 1:**
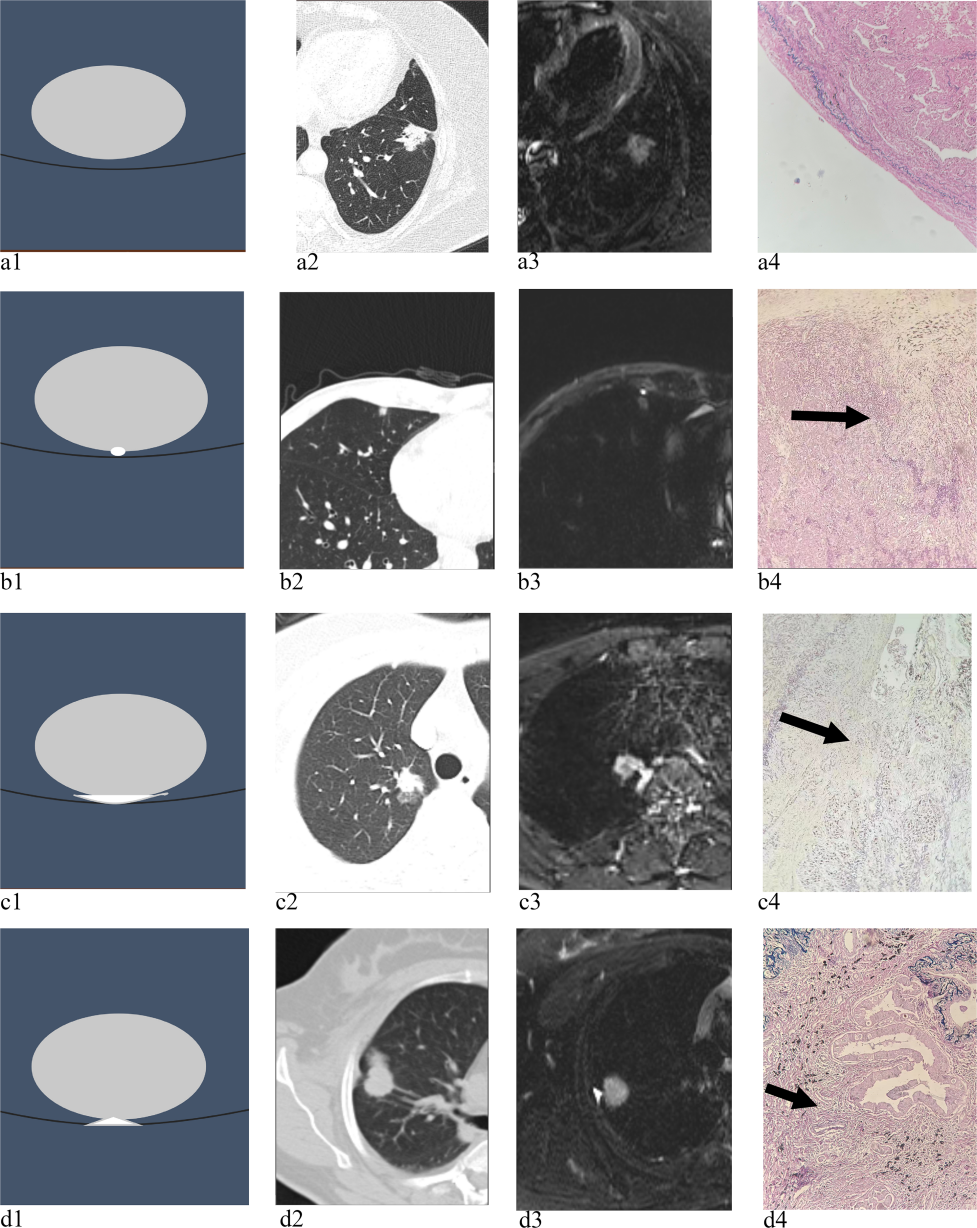
**(a1-a4)** (Grade 0): Absence of T2WI hyperintensity at lesion-pleural interface **(a1)**. 72-year-old female with left lower lobe subpleural solid nodule (26.72 mm). CT demonstrated pleural contact with interlobar fissure involvement and peripheral pleural indentation **(a2)**. MRI revealed Grade 0 signal intensity at lesion-pleural interface **(a3)**. Elastic staining confirmed adenocarcinoma without VPI (×100) **(a4)**. **(b1-b4)** (Grade 1): Focal punctate T2WI hyperintensity at lesion-pleural junction **(b1)**. 58-year-old female with right middle lobe subpleural nodule (8.1 mm). CT showed direct pleural contact **(b2)**. MRI demonstrated Grade 1 interface signal **(b3)**. Elastic staining revealed adenocarcinoma with VPI (×40) (arrow) **(b4)**. **(c1-c4)** (Grade 2): Linear band-like T2WI hyperintensity along pleural contact surface **(c1)**. 64-year-old female with right upper lobe subpleural nodule (16.92 mm). CT confirmed pleural contact **(c2)**. MRI showed Grade 2 interface signal **(c3)**. Elastic staining demonstrated adenocarcinoma with VPI (×100) (arrow) **(c4)**. **(d1-d4)** (Grade 3): Wedge-shaped T2WI hyperintensity at lesion-pleural interface **(d1)**. 59-year-old female with right upper lobe subpleural nodule (23.22 mm). CT revealed pleural retraction signs **(d2)**. MRI displayed Grade 3 interface signal **(d3)**. Elastic staining confirmed adenocarcinoma with VPI (×200) (arrow) **(d4)**.

Two board-certified chest radiologists (Reader 1: 23 years’ experience; Reader 2: 6 years’ experience), blinded to clinical and pathological data, independently conducted consensus interpretation of CT and MRI studies. An experimental design incorporating a 1-month washout period was implemented between CT and MRI interpretation sessions to avoid recall bias. Quantitative analysis included documentation of: a) Tumor maximum axial dimension on MRI, b) nodule type on CT, and c) anatomical localization MRI-based T2WI signal characterization of pleura-contacting lesions was stratified according to the following classification system: Grade 0 (**Figure 1a1**): No evidence of T2WI hyperintensity; Grade 1 (**Figure 1b1**): Focal punctate hyperintensity (≤3 adjacent voxels; 2D area ≤3mm²); Grade 2 (**Figure 1c1**): Linear band-like hyperintensity (length >5mm; length/thickness ratio ≥3:1); Grade 3(**Figure 1d1**): Wedge-shaped hyperintensity (base towards pleura; apex angle <90°). When multiple patterns coexisted, the highest grade was selected (Grade3> Grade2>Grade1> Grade0). To evaluate intraobserver consistency, reader 1 assessed the tumor size and T2WI signal grade on MRI images three months after the initial evaluation again. Any disagreements in the interpretation of imaging features, including signal grades or pleural findings, were resolved in two stages: initial resolution was attempted through direct consensus discussion between readers 1 and 2 while maintaining blinding. For persistently discordant interpretations (n=3 cases), a third senior chest radiologist with 18 years’ experience made the final binding assessment, also blinded to clinical and pathological data. Representative cases are shown in **Figure 1**. All staging classifications presented in this study reflect clinical TNM (cTNM) staging based on preoperative imaging assessment, consistent with the IASLC 8th edition TNM classification system. Pathologic staging (pTNM) was used exclusively as the reference standard for VPI confirmation.

### Statistical analysis

Tumor size measurement agreement was evaluated using the intraclass correlation coefficient (ICC, two-way mixed-effects model for absolute agreement), with interpretation: <0.50 (poor), 0.50-0.75 (moderate), >0.75-0.90 (good), >0.90 (excellent). For T2WI signal grades (ordinal 0-3), weighted kappa (quadratic weights) was computed and interpreted as: ≤0.20 (slight), 0.21-0.40 (fair), 0.41-0.60 (moderate), 0.61-0.80 (substantial), >0.80 (almost perfect). Normality was verified using Shapiro-Wilk tests (α=0.05). Parametric comparisons used independent t-tests with Levene’s test for variance homogeneity; nonparametric comparisons employed Kruskal-Wallis H-tests (Dunn-Bonferroni *post-hoc* adjustments) and Mann-Whitney U tests. Categorical data comparisons used Pearson χ² tests (Fisher’s exact test for cells with expected counts <5). The association between T2WI signal grades (0–3) and nodule type (categorical: solid vs. part-solid) was evaluated using Pearson’s chi-square test for independence. Diagnostic metrics (sensitivity, specificity, PPV, NPV, accuracy) were calculated with simple presence or absence of each grade separately. Likelihood ratios (LRs) were classified per evidence-based criteria: Positive LR: >10 (strong), 5-10 (moderate), 2-5 (weak), 1-2 (minimal), Negative LR: 0.5-1 (minimal rule-out), 0.2-0.5 (weak), 0.1-0.2 (moderate), <0.1 (strong). Furthermore, to determine the significant factors associated with VPI, we performed binomial logistic regression analysis. Independent variables, including age, sex, tumor size and type of visceral signal were included in the model by using forced entry approach. Based on the logistic regression results, a column chart was established and its ROC curve was plotted. Prediction performance was evaluated using: Decision curve analysis (DCA). Net benefit across 0-100% threshold probabilities Calibration curves: B-spline fitting with 200 bootstrap resamples. Analyses were performed in R v4.2.3. Statistical significance: two-tailed α=0.05.

## Results

**Figure 2:**
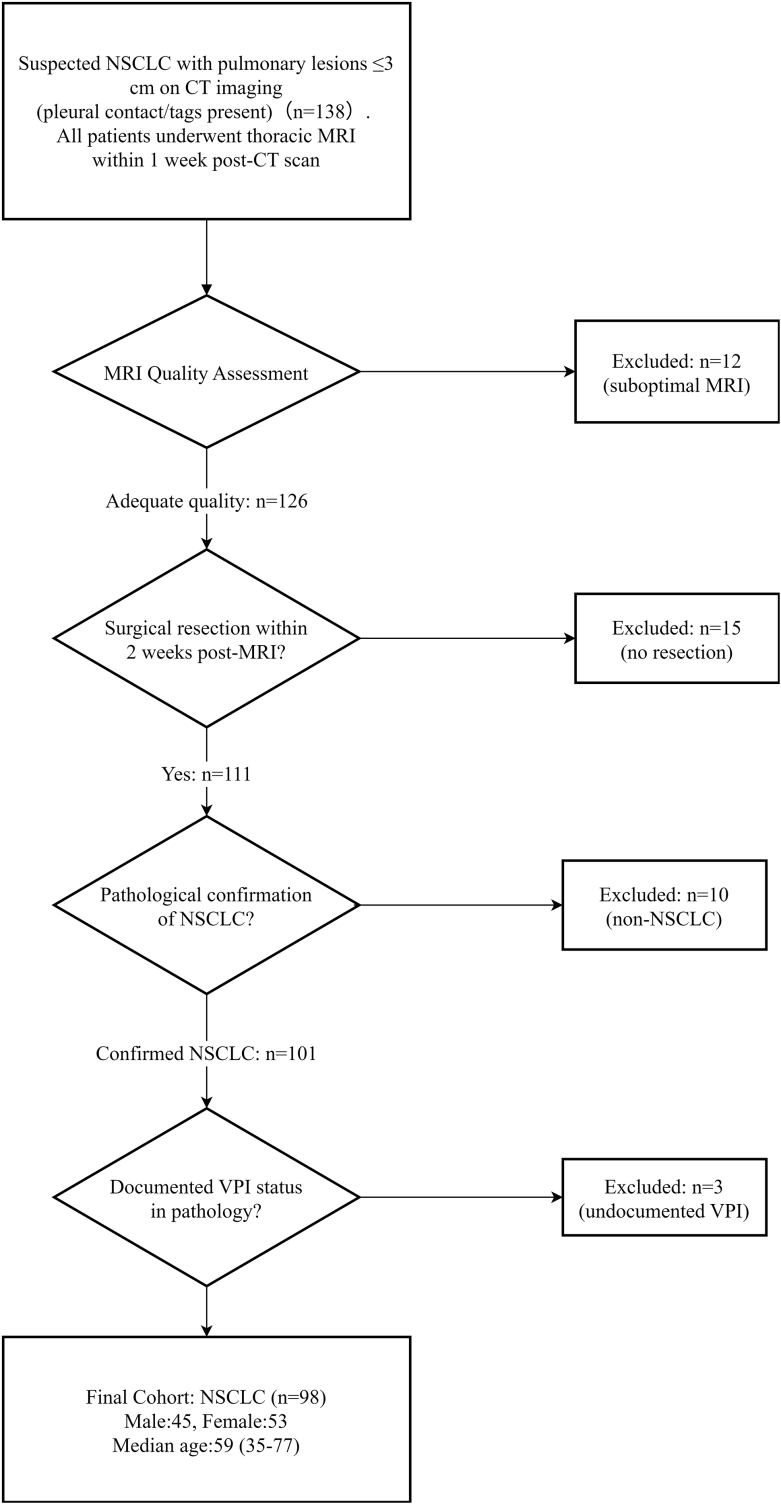
Flowchart illustrating patient selection. NSCLC, non-small cell lung cancer.

From the initial 138 enrolled patients, 40 were excluded through sequential application of criteria: (1) 12 cases were excluded due to objective diagnostic limitations: severe respiratory motion (n=9) or susceptibility artifacts (n=3) prohibiting clear delineation of the tumor-pleura interface, (2) 15 patients were excluded for not undergoing surgical resection of pulmonary lesions within 2 weeks after MRI scan, (3) 10 cases were excluded postoperatively due to non-NSCLC pathological confirmation, (4) 3 additional cases were excluded owing to undocumented VPI status in pathological reports, because their visceral pleura was significantly damaged during surgery (e.g., due to adhesiolysis or staple line placement at the tumor-pleural interface). A final cohort of 98 NSCLC patients (45 males,53 females; median age 59 years, range 35-77) with lesions ≤3 cm was retained for analysis (**Figure 2**). Baseline features of excluded and included cases are presented in **Supplementary Table S2**.

The study cohort comprised 98 patients with NSCLC ≤3 cm (mean age 59.4 ± 10.20 years; 45 males). Invasive adenocarcinoma was the predominant histologic type (73.5%, 72/98), followed by squamous cell carcinoma (19.4%, 19/98). Interobserver and intraobserver agreement for quantitative and qualitative measurements was robust. Tumor size demonstrated near-perfect reliability (interobserver ICC = 0.977, 95% CI: 0.961–0.987; intraobserver ICC = 0.987, 95% CI: 0.980–0.992). T2WI signal grade also achieved excellent consistency (interobserver κ = 0.915, 95% CI: 0.873–0.945; intraobserver κ = 0.972, 95% CI: 0.956–0.983). The cohort demonstrated a mean tumor size of 18.86 ± 5.56 mm, predominantly classified as cT1b (49/98) or cT1c (43/98) (**Table 1**).

**Table 1:** Clinical Features of 98 patients with NSCLC.

Parameter	Result
Age(y) *	59.4 ± 10.20 (35–77)
males (45)	57.11 ± 10.60(35-75)
females (53)	61.22 ± 9.8(36-77)
Tumor size(mm)	18.86 ± 5.56
Pathology	
Invasive adenocarcinoma	72
Mucinous Adenocarcinoma	3
Squamous cell carcinoma	19
Adenosquamous carcinoma	4
cT stage	
cT1a	6
cT1b	49
cT1c	43
cN stage	
cN0	77
cN1	14
cN2	7
Location	
RUL	21
RML	19
RLL	14
LUL	28
LLL	16

*TNM classifications represent clinical staging (cTNM) determined through preoperative imaging assessment. Pathologic staging (pTNM) was used solely for VPI validation. Data are means ± standard deviation. Data in parentheses are range. LLL, left lower lobe; LUL, left upper lobe; RLL, right lower lobe; RML, right middle lobe; RUL, right upper lobe.

Comparisons between VPI-positive (n=35) and VPI-negative (n=63) groups revealed significantly larger tumor size in the VPI-positive cohort (22.32 ± 5.58 mm vs. 16.17 ± 4.22 mm, *P* = 0.024). A higher proportion of VPI-positive lesions exhibited T2WI hyperintensity (85.71% vs. 52.38%; *P* = 0.001), with T2WI signal grade distribution differing markedly between groups (*P* < 0.001). Grade 3 signal predominated in VPI-positive cases (37.1%, 13/35), whereas grade 0 was most frequent in VPI-negative lesions (47.6%, 30/63) (**Table 2**). Among 63 CT-suspected but pathologically VPI-negative cases, 47.6% (30/63) showed Grade 0 T2WI signal that could have correctly downstaged these patients.

**Table 2:** Comparison of clinical-pathologic features in 98 patients with NSCLC with and without VPI.

Clinical-pathologic features	Absent VPI	VPI	*P* value
No. of Patients	63	35	
Sex			0.650
Men	33	15	
Women	30	20	
Age	60.66 ± 9.44	56.84 ± 10.00	0.071
Tumor histologic analysis			0.396
Invasive adenocarcinoma	45	27	
Mucinous adenocarcinoma	3	0	
Squamous cell carcinoma	12	7	
Adenosquamous carcinoma	3	1	
Tumor size (mm)	16.17 ± 4.22	22.32 ± 5.58	0.024
Nodule type			0.630
Solid	31	19	
Part-Solid	32	16	
Presence of T2WI hyperintensity	52.38% (33/63)	85.71% (30/35)	0.001
T2WI signal grade			<0.001
Grade 0	30	5	
Grade 1	19	7	
Grade 2	10	10	
Grade 3	4	13	

The differential distribution of T2WI signal grades demonstrated significant associations with VPI status across nodule types (*P* < 0.01). Among solid nodules, Grade 3 signals exhibited the strongest VPI correlation (42.11%, 8/19), compared to Grade 0-1. Conversely, Grade 0 signals predominated in VPI-negative solid nodules (32.3%, 10/31). Part-solid nodules showed analogous graded relationships - Grade 3 maintained 31.25% VPI probability (5/16), with Grade 0 signals again being protective (12.50% for VPI) (**Table 3**).

**Table 3:** Association between T2WI signal grades and nodule type by VPI status.

Nodule type	T2WI signal grade	Absent VPI	VPI	*P* value
Solid	Grade 0	10	3	0.007
	Grade 1	11	2	
	Grade 2	8	6	
	Grade 3	2	8	
Part-Solid	Grade 0	20	2	0.002
	Grade 1	8	5	
	Grade 2	2	4	
	Grade 3	2	5	

T2WI signal grade demonstrated incremental diagnostic utility for VPI prediction. As illustrated in **Figure 1**, grade 3 achieved the highest specificity (93.65%, 95%CI 84.48–98.19%), positive predictive value (76.47%, 95%CI 50.11–93.19%), and LR+ (5.85, 95%CI 2.35–14.59), with an accuracy of 73.47% (95%CI 63.58–81.84%), corresponding to typical histopathologic features of pleural invasion. This indicates that when Grade 3 is observed, the probability of VPI increases approximately sixfold compared to baseline, constituting a strong diagnostic indicator per evidence-based thresholds (LR+ >5). The overall model combining all T2WI signal grades yielded 85.71% (95%CI 69.74–95.19%) sensitivity and 47.62% (95%CI 34.86–60.58%) specificity (**Table 4**).

**Table 4:** Diagnostic statistics of pleural T2WI signal grade to predict VPI.

T2WI signal grade	Case no.	Sensitivity (95%CI)	Specificity (95%CI)	PPV (95%CI)	NPV (95%CI)	Accuracy (95%CI)	LR+(95%CI)
Grade 1	26	20.00% (8.07–39.74%)	69.84% (56.56–80.84%)	26.92% (11.57–49.81%)	61.11% (48.47–72.62%)	52.04% (41.67–62.27%)	0.66 (0.28–1.53)
Grade 2	20	28.57% (14.64–47.60%)	84.13% (72.73–91.92%)	50.00% (27.20–72.80%)	67.95% (55.38–78.69%)	64.29% (53.86–73.70%)	1.80 (0.82–3.94)
Grade 3	17	37.14% (21.48–55.85%)	93.65% (84.48–98.19%)	76.47% (50.11–93.19%)	72.84% (60.88–82.69%)	73.47% (63.58–81.84%)	5.85 (2.35–14.59)
Grade 1-3	63	85.71% (69.74–95.19%)	47.62% (34.86–60.58%)	47.62% (34.86–60.58%)	85.71% (69.74–95.19%)	61.22% (50.81–70.88%)	1.64 (1.23–2.18)

LR+ = positive likelihood ratio. LR+ interpretation: >10 (strong), 5-10 (moderate), 2-5 (weak), 1-2 (minimal).

Multivariate logistic regression identified tumor size (OR = 1.126 per mm, 95% CI:1.005–1.276, *P* = 0.049) and elevated T2WI signal grades as independent predictors of VPI. Grade 2 (OR = 10.533, 95% CI:2.529–52.711, *P* = 0.002) and grade 3 (OR = 46.379, 95% CI:8.581–359.619, *P* < 0.001) demonstrated robust associations with VPI risk (**Table 5**).

**Table 5:** Logistic regression results of independent variables.

Predictor	Estimate	SE	Z	*P*	Odds Ratio	Lower	Upper
(Intercept)	-6.059	2.196	-2.759	0.006	0.002	0.000	0.128
age	0.029	0.027	1.063	0.288	1.029	0.977	1.088
Tumor size (mm)	0.126	0.056	2.249	0.024	1.134	1.021	1.276
Sex (M)	-0.262	0.572	-0.457	0.647	0.770	0.243	2.35
Nodule type (Solid)	0.522	0.564	0.926	0.354	1.686	0.565	5.277
T2WI signal grade 1	0.754	0.694	1.087	0.277	2.126	0.549	8.715
T2WI signal grade 2	2.178	0.723	3.012	0.003	8.826	2.265	40.057
T2WI signal grade 3	3.398	0.854	3.98	0.000	29.890	6.374	191.869

**Figure 3:**
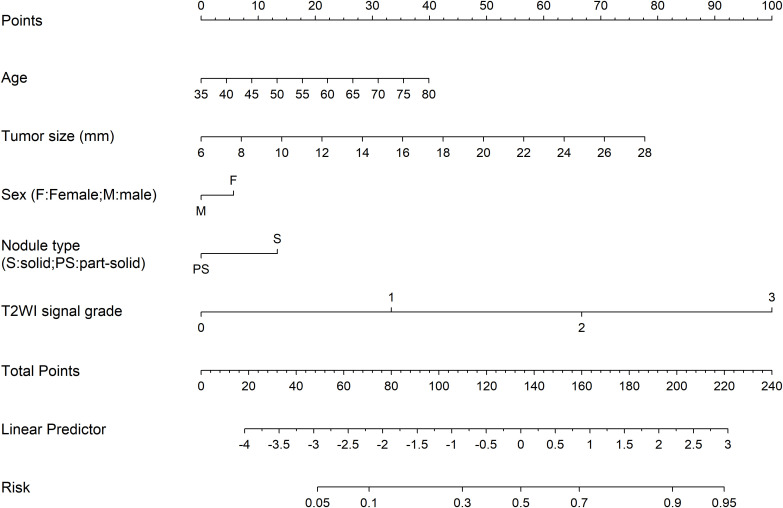
A column chart was drawn based on age, tumor size, gender, nodule type and T2WI signal grade.

**Figure 4:**
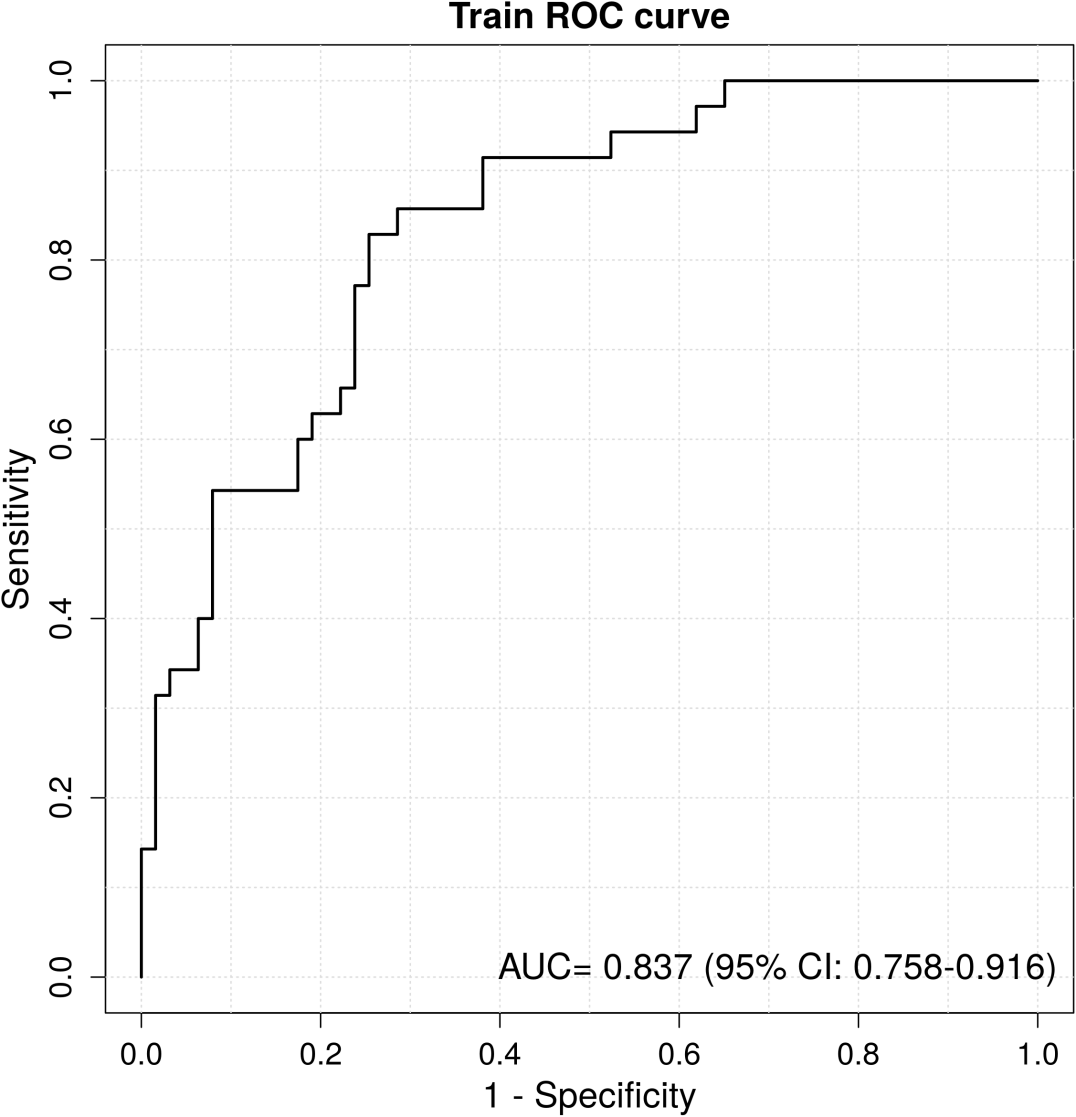
The ROC curve of the column chart. The AUC value of 0.837(95% CI 0.758-0.916).

**Figure 5:**
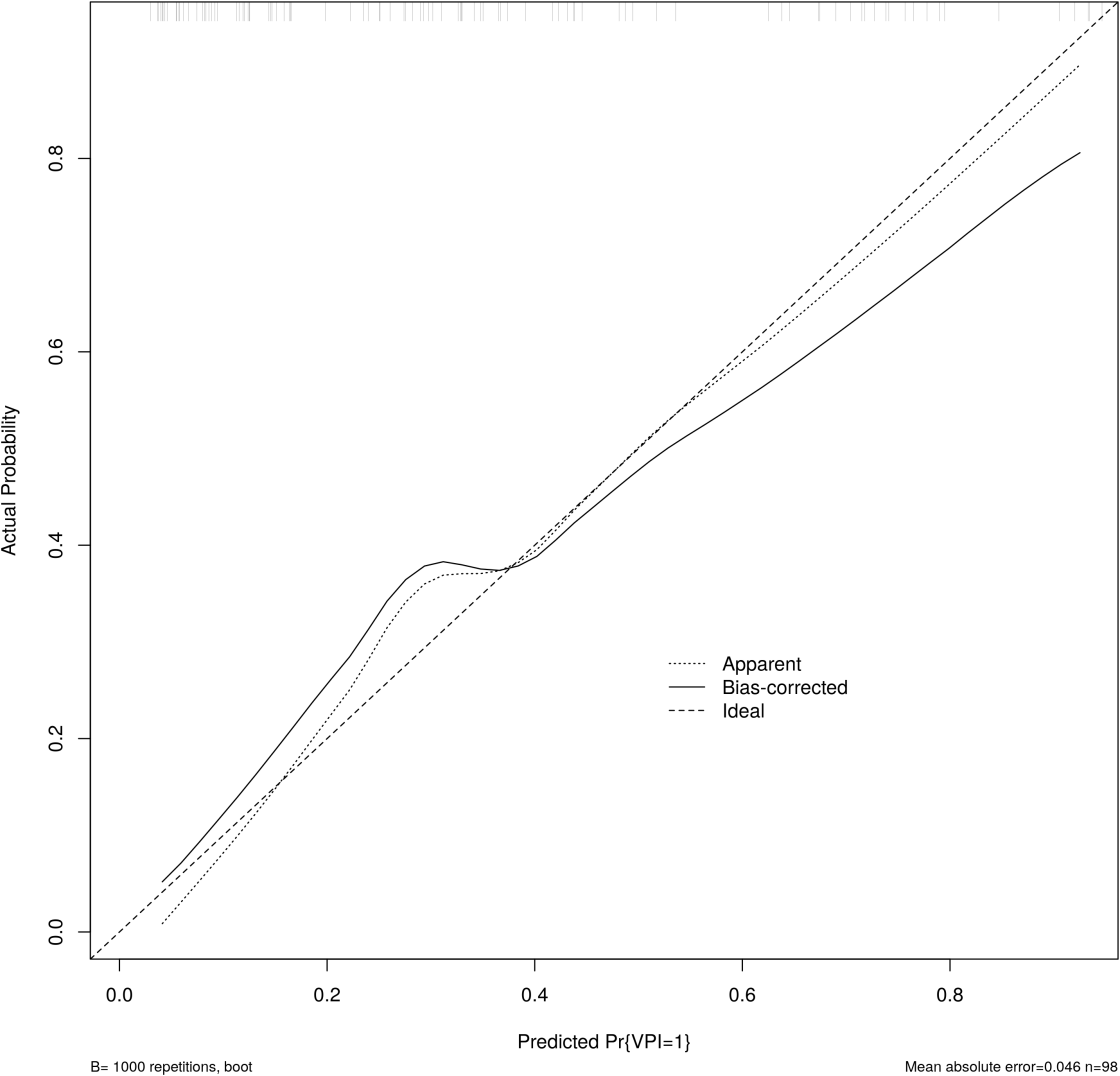
Calibration curves illustrated a strong linear relationship between the predicted probabilities and actual occurrence probabilities.

**Figure 6:**
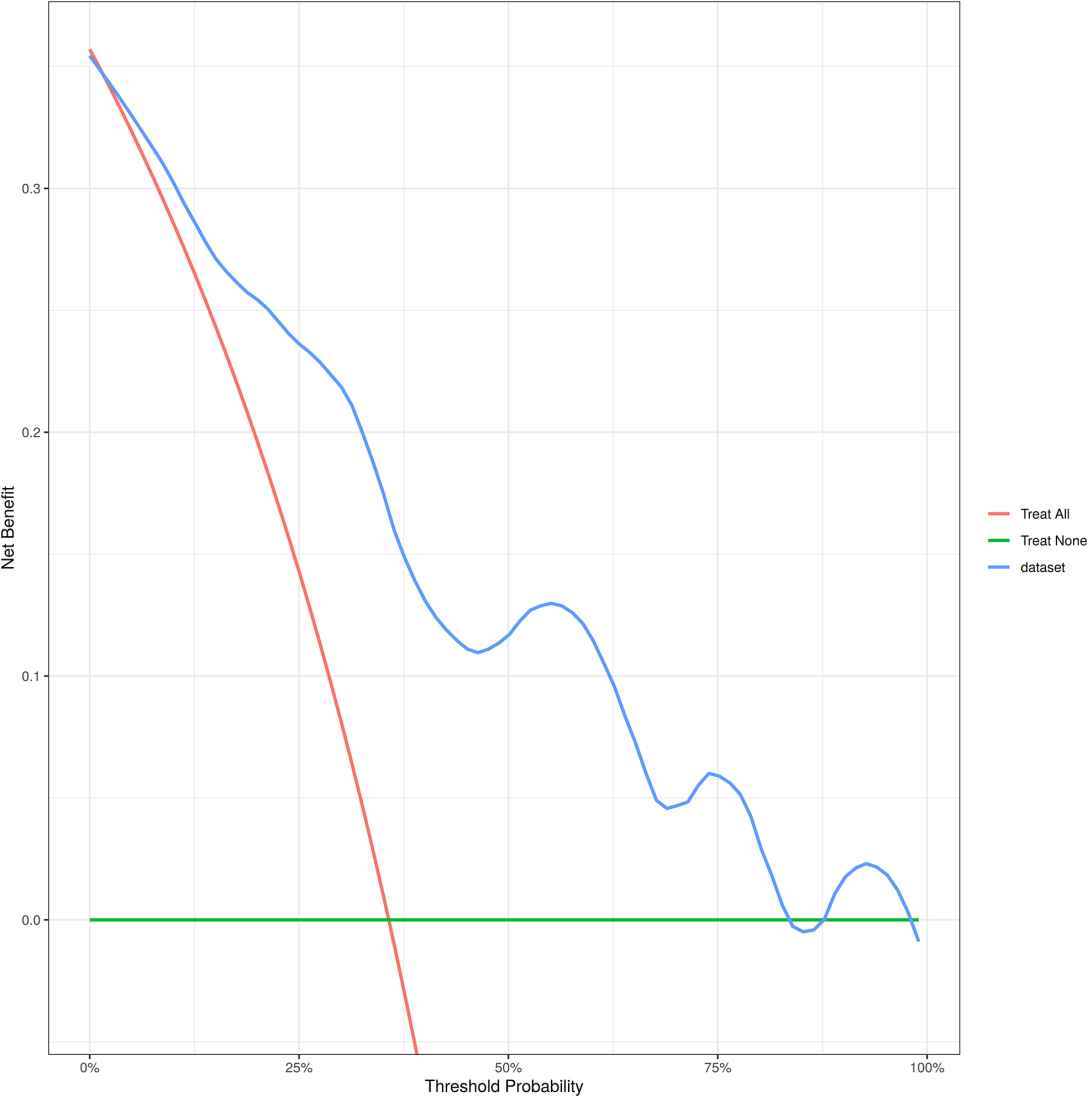
DCA curve of column chart.

A column chart was drawn based on age, tumor size, gender, nodule type and T2WI signal grade (**Figure 3**). ROC curve analysis demonstrated that the AUC for the established model was 0.837 (95% CI 0.758-0.916), indicating good discrimination capability (**Figure 4**). Calibration curves illustrated a strong linear relationship between the predicted probabilities and actual occurrence probabilities, with bias-corrected predictions aligning closely with ideal scenarios (**Figure 5**). DCA analysis revealed the model’s net benefit surpassed default strategies across threshold probabilities of 5–84%. This suggests clinical value when VPI probability thresholds align with T-upstaging or adjuvant therapy considerations (**Figure 6**).

## Discussion

This study establishes the diagnostic utility of T2WI in predicting VPI in NSCLC ≤3 cm, demonstrating that distinct T2WI signal patterns at the lesion-pleural interface correlate strongly with histopathologic VPI status. Our novel four-tier grading system (Grades 0–3)—based on progressive T2WI hyperintensity patterns—achieved high specificity (93.65%) and positive predictive value (76.47%) for Grade 3 lesions, significantly outperforming traditional CT-based criteria. Combining tumor size with T2WI signal grades yielded a robust predictive model (AUC = 0.837), which synergizes radiomic and anatomic parameters to refine preoperative VPI assessment. The DCA further supports clinical adoption, demonstrating that our model reduces unnecessary aggressive interventions without omitting high-risk cases. This balances oncologic rigor against overtreatment risks—a critical consideration given VPI’s prognostic impact. The study expands upon limited existing MRI literature by systematically validating T2WI signal characteristics as predictors of VPI in early-stage NSCLC. While prior works (19) compared MRI and CT for pleural contact assessment, our graded T2WI system provides novel stratification of invasion likelihood. In the absent VPI group, 47.6% showed grade0 signal, but about 52.38% of the cases still had T2WI hyperintensity (grade1-3), which may reflect that other benign pleural reactions were not controlled (such as inflammation or fibrosis). Future studies could employ: (1) Multiparametric MRI combining DWI (restricted diffusion in tumors vs facilitated in inflammation) and dynamic contrast enhancement (washout vs persistent patterns); (2) PET-MRI fusion assessing FDG avidity discordance; (3) Radiomics correlation with serum markers (e.g., IL-6, CRP levels).

Existing methods for preoperative VPI prediction predominantly rely on CT features. Hsu (17) retrospective analysis on 141 cases of NSCLC without pleural contact, type 2 pleural tags may ultimately be a useful CT finding to increase the accuracy and specificity of early diagnosis for VP in NSCLC that does not abut the pleura, which accuracy was 71%, sensitivity was 36.4, specificity was 92.8, PPV was 76.2%, NPV was 69.6%, LR + 5.06. HSU (15) discussed the effectiveness of the tumor edge morphology of peripheral NSCLC in CT images as a diagnostic indicator of pleural invasion, and compared its differences with traditional CT indicators (obtuse angle, pleural contact length >3cm, pleural thickening).Tumor border type 5 has a high PPV and high specificity for predicting pleural invasion by peripheral NSCLC. Cord-like pleural tags (20) or “bridge tag sign” (16) have also been associated to VPI. The proposed T2WI grading system exhibited significantly superior specificity in VPI detection, attributable to MRI’s intrinsic advantages: (1) superior soft-tissue discrimination enabling precise tumor-pleura margin delineation (**Figure 1d3** vs **Figure 1d2**); (2) Direct visualization of microstructural disruption through T2 signal alterations reflecting edema or neovascularization (**Figure 1d4**); (3) While CT relies on secondary morphological signs (pleural tags/thickening), our Grade 3 criteria detect actual pathophysiological changes at the elastic layer level.

While CT retains diagnostic utility, the high PPV(44% -56%) demonstrates that there is insufficient evidence for clinical staging (cT1-cT2) (18). Furthermore (21), agreement among thoracic radiologists was only fair to moderate and the diagnostic performance of radiological evaluation for the diagnosis of VPI was far from being clinically reliable, with positive predictive values (PPV) ranging between 40.2 and 57.1% for solid lesions, and even a lower range for part-solid lesions. The clinical implementation of preoperative MRI for VPI assessment necessitates careful consideration of its targeted applications. First, MRI demonstrates particular utility in resolving diagnostic uncertainty when CT reveals pleural contact or tags but presents conflicting risk features, such as sub-2 cm lesions with linear pleural tags versus larger tumors exhibiting obtuse angles. The superior predictive performance of MRI is evidenced by Grade 3 T2WI signals achieving a positive predictive value of 76.47%, representing a significant improvement over CT’s established range of 44-56%. Second, preoperative MRI proves invaluable for surgical planning in clinically complex scenarios, particularly when evaluating candidates for sublobar resection among elderly patients or those with compromised pulmonary function. The technique’s 93.65% specificity substantially reduces both false-positive driven unnecessary lobectomies and false-negative associated incomplete resections. Furthermore, MRI offers distinct advantages for radiation-sensitive populations, including younger patients requiring serial imaging surveillance for multifocal nodules, by eliminating cumulative ionizing radiation exposure. These findings collectively position MRI not as a routine replacement for CT, but rather as a specialized diagnostic adjunct precisely indicated when conventional imaging yields equivocal results that substantially impact therapeutic decision-making. The economic justification emerges from the calculation that prevention of a single unnecessary lobectomy offsets the cost of approximately 71 MRI examinations, while simultaneously preserving pulmonary function and optimizing oncologic outcomes through more accurate cT-staging. While the study primarily validates the standalone feasibility of T2WI grading, the observed 93.65% specificity of Grade 3 signals (compared to CT’s 44-56% PPV) shows strong potential for reducing false-positive diagnoses when MRI is applied to CT-indeterminate cases. In particular, the absence of T2WI hyperintensity (Grade 0) in nearly half of CT-suspected but VPI-negative cases suggests a possible role for MRI in de-escalating unnecessary surgical interventions. However, we emphasize that definitive demonstration of incremental value requires future studies specifically designed to perform head-to-head comparison with standardized CT criteria.

At present, there are few studies on the prediction of VPI of NSCLC by MRI. Zhang (19)revealed no significant differences between the diagnostic accuracies of CT and MRI for evaluating the contact length, angle of mass margin, or arch distance-to-maximum tumor diameter ratio as predictors of VPI. The advantage of MRI is its clear depiction of the tumor-pleura interface margin, facilitating VPI detection. While MRI’s inherent advantage in differentiating tumor infiltration from benign pleural reactions has been theorized, prior studies focused predominantly on CT metrics. Our findings extend this paradigm by demonstrating that T2WI’s spatial resolution and contrast facilitate precise identification of VPI-associated pleural microstructural disruption. Particularly, Grade 3 wedge-shaped hyperintensity—likely reflecting localized pleural edema and neovascular proliferation from tumor invasion—achieved a 5.85-fold higher LR+ than CT-based criteria, underscoring its potential to reduce overstaging. Ryu (22) demonstrates minimal pleural effusion(PE) is a commonly encountered clinical concern in staging NSCLCs. Its presence is an important prognostic factor of worse survival, especially in early-stage disease. Hyperintensity in T2WI may represent minimal PE. Part-solid nodules were also included in this study. Ahn et al. (23) found that the CT findings associated to VPI in part-solid nodules were pleural contact, pleural thickening, solid proportion greater than 50%, and nodule size greater than 20 mm, although all of them with a low PPV of less than 40%. Our findings demonstrate that higher T2WI signal grades correlate with more solid tumor components, suggesting that pleural mechanical stress from denser tumors may predispose to both VPI and peritumoral reactive changes visible on MRI. This aligns with Heidinger et al.’s CT-based observation (24), though our model provides superior specificity through direct assessment of pleural microstructural disruption. Furthermore, our model’s integration of tumor size and T2WI signal grades aligns with emerging evidence that VPI risk escalates with lesion dimensions and distinct radiologic signatures, addressing a critical gap in purely morphology-driven CT assessments. While the current TNM staging system appropriately prioritizes measurement of the solid/invasive component diameter for cT categorization, our T2WI grading system provides orthogonal stratification by evaluating the tumor-pleura interface biology. Notably, among part-solid nodules in our cohort (n=48), higher T2WI grades still showed significant VPI prediction. This suggests MRI signal characteristics capture distinct pathophysiological information beyond morphologic size criteria. Clinically, integrating both parameters - invasive size for baseline cT staging and T2WI grading for VPI likelihood - could enable more comprehensive risk assessment, particularly when considering sublobar resection eligibility or adjuvant therapy decisions.

Several limitations warrant consideration. Firstly, the single-center design and modest sample size (n=98) may limit generalizability. External or temporal validation was need. The T2WI grading system requires standardization across institutions to ensure reproducibility, as qualitative signal interpretation may vary among radiologists. Future multicenter validation is essential to confirm our findings’ external validity. To address the single-center limitation, we propose a three-phase validation strategy: (1) Protocol standardization across 3–5 tertiary centers using customized MRI phantoms; (2) Radiologist training programs incorporating our online grading module with 50 reference cases; (3) Prospective enrollment targeting 300 cases with dual-institution histopathology review. Secondly, although respiratory-triggered MRI reduced motion artifacts, subtle pleural architectural distortions in subcentimeter lesions may still compromise signal grading accuracy. Consequently, 12 cases were excluded due to suboptimal image quality. Future studies should develop more advanced techniques to further optimize thoracic MRI acquisitions. Furthermore, patients with non-NSCLC pathology and those with inadequate MRI quality, who could not be preoperatively distinguished from included cases, were excluded from the primary analysis. This may have led to an overestimation of the diagnostic performance of T2WI-based assessment. Thirdly, the study did not evaluate complementary MRI sequences (e.g., dynamic contrast-enhanced magnetic resonance imaging (DCE-MRI) or DWI-derived ADC values), which may further enhance VPI prediction. Beyond T2WI, we highlight DCE-MRI as a priority investigation target. Pilot data (25) suggest quantitative parameters (Ktrans, Kep or ve) at tumor may distinguish malignant from benign nodules as well as differentiate different types of malignancies. A multicenter study evaluating combined T2WI+DCE-MRI is currently in protocol development. Fourthly, the pathological data primarily documented VPI as a binary outcome (present/absent) according to surgical reporting standards. This precluded statistical mapping between imaging grades and depth-specific invasion levels. Future studies incorporating systematic PL subtyping should explore this relationship to determine whether advanced T2WI signal patterns (e.g., Grade 3) preferentially indicate full-thickness pleural infiltration (PL2/3). Lastly, selection bias may exist since only patients with CT-suspected pleural contact were included, excluding peripherally located tumors without initial pleural involvement signs. Because the number of VPI-positive events was limited relative to the number of predictors, the multivariable model may be prone to overfitting, and its coefficients and apparent performance should be interpreted with caution until validated in larger, multicenter cohorts. Future multicenter studies incorporating multimodal MRI metrics and machine-learning algorithms are warranted to optimize VPI prediction frameworks.

## Data Availability

The raw data supporting the conclusions of this article will be made available by the authors, without undue reservation.
