# Supplementary material for: A T2-weighted MRI grading system for preoperative prediction of visceral pleural invasion in small non-small cell lung cancers
Source: Front Oncol. 2025 Nov 27;15:1682929. doi: 10.3389/fonc.2025.1682929 (PMC12695614; doi:10.3389/fonc.2025.1682929)
Supplement: Supplementary file 1 [file Table1.docx]

Supplementary Table S1. STARD checklist

| Section | No | Item | Reported on Section/Paragraph | Reported on Line Number | Manuscript Content Excerpt |
| --- | --- | --- | --- | --- | --- |
| TITLE OR ABSTRACT |  |  |  |  |  |
|  | 1 | Identification as a study of diagnostic accuracy using at least one measure of accuracy (such as sensitivity, specificity, predictive values or AUC) | Title Page / Abstract | Results | *Grade 3 T2WI signals (wedge-shaped) achieved 93.65% specificity and 76.47% PPV for VPI* |
| ABSTRACT |  |  |  |  |  |
|  | 2 | Structured summary of study design, methods, results and conclusions | Abstract | Abstract | Structured format (Purpose, Methods, Results, Conclusion) |
| INTRODUCTION |  |  |  |  |  |
|  | 3 | Scientific and clinical background, including the intended use and clinical role of the index test | Introduction / Paragraphs 1-3 | Line 49-82 | Background on VPI significance, CT limitations, MRI rationale |
|  | 4 | Study objectives and hypotheses | Introduction / Paragraph 3 | Line 79-82 | *Therefore, the purpose of this study……* |
| METHODS |  |  |  |  |  |
| Study design | 5 | Whether data collection was planned before the index test and reference standard were performed (prospective study) or after (retrospective study) | MATERIALS AND METHODS / Paragraph 1 | Line 85 | This prospective single-center study... |
| Participants | 6 | Eligibility criteria | Study Participants/ Paragraph 2 | Line 95 | *The study initially enrolled 138 patients with…extending from nodule to pleura* |
|  | 7 | On what basis potentially eligible participants were identified (such as symptoms, results from previous tests, inclusion in registry) | Study Participants/ Paragraph 2 | Line 96 | Identified by CT pleural contact/tags |
|  | 8 | Where and when potentially eligible participants were identified (setting, location and dates) | Study Participants/ Paragraph 2/ Figure 1 | Line 85、97, Figure 1 | *at the Forth Hospital of Hebei Medical University*  *from January 2021 to March 2023* |
|  | 9 | Whether participants formed a consecutive, random or convenience series | Study Participants | Line 96 | randomly |
| Test methods | 10a | Index test, in sufficient detail to allow replication | CT Scanning- Imaging Analysis | Line103-164 | CT/MRI protocols and 4-tier T2WI grading system |
|  | 10b | Reference standard, in sufficient detail to allow replication | Pathological Analysis/ Paragraph 1 | Line135-141 | PL0-3 classification via elastic staining |
|  | 11 | Rationale for choosing the reference standard (if alternatives exist) | N/A | N/A | Histopathology as gold standard for VPI |
|  | 12a | Definition of and rationale for test positivity cut-offs or result categories of the index test, distinguishing prespecified from exploratory | Imaging Analysis / Paragraph 1 | Line147-155 | Prespecified T2WI grades 0-3 with pictorial definitions |
|  | 12b | Definition of and rationale for test positivity cut-offs or result categories of the reference standard, distinguishing prespecified from exploratory | Pathological Analysis/ Paragraph 1 | Line135 | PL0-3 criteria per 8th TNM edition |
|  | 13a | Whether clinical information and reference standard results were available to the performers or readers of the index test | Imaging Analysis / Paragraph 1 | Line144 | Blinded radiologists without pathology access |
|  | 13b | Whether clinical information and index test results were available to the assessors of the reference standard | Imaging Analysis / Paragraph 1 | Line144, 146 | Radiologists blinded to pathology; 1-month washout period |
| Analysis | 14 | Methods for estimating or comparing measures of diagnostic accuracy | Statistical Analysis / Paragraph 1 | Line182 | Logistic regression, ROC/DCA analysis |
|  | 15 | How indeterminate index test or reference standard results were handled | Imaging Analysis/ Paragraph 1 | Line161 | Third-reader arbitration for discordant cases |
|  | 16 | How missing data on the index test and reference standard were handled | Study Participants / Paragraph 1 | Line100 | Excluded cases with missing data documented |
|  | 17 | Any analyses of variability in diagnostic accuracy, distinguishing prespecified from exploratory | Results / Tables 2-3 | Results (Comparative Analysis) | Subgroup analyses by tumor size/signal grade |
|  | 18 | Intended sample size and how it was determined | Results/ Paragraph 1 | Line 201 | Sample size justified by feasibility (138 screened →98 analyzed) |
| RESULTS |  |  |  |  |  |
| Participants | 19 | Flow of participants, using a diagram | Figure 1 | Results (Participant Flow) | CONSORT-style enrollment diagram |
|  | 20 | Baseline demographic and clinical characteristics of participants | Results / Paragraph 2/Table 1 | Line 205 | Demographics, tumor features |
|  | 21a | Distribution of severity of disease in those with the target condition | Results / Paragraph 3/Table 2 | Line 215 | VPI+ group tumor size/signal distribution |
|  | 21b | Distribution of alternative diagnoses in those without the target condition | Results / Table 2 | Results (Comparative Analysis) | VPI- group characteristics |
|  | 22 | Time interval and any clinical interventions between index test and reference standard | Study Participants Paragraph 1 | Line 101 | MRI→surgery within 2 weeks |
| Test results | 23 | Cross tabulation of the index test results (or their distribution) by the results of the reference standard | Results / Tables 2-3 | Results (Diagnostic Statistics) | Cross-tabulation of T2WI grades vs VPI status |
|  | 24 | Estimates of diagnostic accuracy and their precision (such as 95% CIs) | Results / Tables 3-4; Figure 4 | Results (Diagnostic Performance) | AUC=0.837 (95%CI:0.758-0.916) |
|  | 25 | Any adverse events from performing the index test or the reference standard | N/A | N/A | No adverse events reported |
| DISCUSSION |  |  |  |  |  |
|  | 26 | Study limitations, including sources of potential bias, statistical uncertainty and generalisability | Discussion / Paragraph 5 | Line 335 | *Several limitations warrant consideration……* |
|  | 27 | Implications for practice, including the intended use and clinical role of the index test | Discussion / Paragraph 3 | Line 300 | Potential for preoperative T-upstaging decisions |
| OTHER INFORMATION |  |  |  |  |  |
|  | 28 | Registration number and name of registry | N/A | N/A | Not explicitly reported |
|  | 29 | Where the full study protocol can be accessed | N/A | N/A | Protocol availability not stated |
|  | 30 | Sources of funding and other support; role of funders | Funding information | Funding information | This work was supported by the Medical Science Research Project of Hebei [grant numbers 20230789] |

Supplementary Table S2 Baseline features of excluded vs included cases

| Features |  | Included (n=98) | Excluded (n=40) | *P* value |
| --- | --- | --- | --- | --- |
| Age |  | 59.4±10.2 | 58.2±9.8 | 0.312 |
| Female Sex |  | 54% | 52% | 0.587 |
| Tumor Size |  | 18.9±5.6 mm | 16.8±5.1 mm | 0.078 |
